# High serum exosomal long non‐coding RNA DANCR expression confers poor prognosis in patients with breast cancer

**DOI:** 10.1002/jcla.24186

**Published:** 2022-02-11

**Authors:** Wenbiao Shi, Xiaoyan Jin, Yuanfan Wang, Qiang Zhang, Linjun Yang

**Affiliations:** ^1^ Department of Surgical Oncology Taizhou Municipal Hospital Taizhou City Zhejiang Province China

**Keywords:** breast cancer, diagnosis, exosome, lncDANCR, prognosis

## Abstract

**Background:**

Exosomal long non‐coding RNAs (lncRNAs) serve as excellent candidate biomarkers for clinical applications. The expression of differentiation antagonizing non‐protein coding RNA (DANCR) has been shown to be decreased in breast cancer (BC) tissues and cell lines. However, the clinical value of circulating exosomal DANCR in BC has not been explored.

**Methods:**

A total of 120 BC patients, 70 benign breast disease (BBD) patients, and 105 healthy women were recruited in this study. Total RNA was extracted from serum samples, and the level of serum exosomal lncRNA DANCR was evaluated by quantitative real‐time reverse transcription‐polymerase chain reaction (qRT‐PCR).

**Results:**

Serum exosomal lncRNA DANCR levels were significantly higher in BC patients than in BBD patients and normal controls. The diagnostic performance of serum exosomal lncRNA DANCR was good, and the combination of serum exosomal lncRNA DANCR, CA153, and CEA greatly improved the diagnostic accuracy for BC. High serum exosomal lncRNA DANCR level was associated with various clinicopathological variables including lymph node metastasis, ER status, HER2 status, and TNM stage. In addition, the BC patients in the high serum exosomal lncRNA DANCR expression group had significantly shorter 5‐year overall survival time. Multivariate analysis demonstrated that serum exosomal lncRNA DANCR was an independent risk factor for BC.

**Conclusion:**

Serum exosomal lncRNA DANCR may be a useful non‐invasive biomarker for the clinical diagnosis and prognosis of BC.

## Graphical abstract

Receiver operating characteristic curves of single and combining three markers with the optimum cut‐off values in differentiating BC from controls.

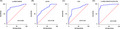

## 1 INTRODUCTION

Breast cancer (BC) is the most frequent cancer among women around the world.^1^, ^2^ In China, more than 272,400 newly diagnosed BC were reported, causing about 70,700 deaths in 2015. BC alone accounts for about 15% of all new cancers in women.^3^, ^4^ Despite recent advances in surgical techniques and adjuvant chemotherapy, the clinical outcome of BC patients remains unfavorable due to the recurrence and metastasis.^5^ Early diagnosis and treatment significantly improve the prognosis of this malignancy. Cancer antigen 153 (CA153) and carcinoembryonic antigen (CEA) are the most commonly used biomarkers for BC. However, diagnostic and prognostic accuracy of CA153 and CEA is poor. Therefore, it is urgent to identify novel, reliable biomarkers for GC diagnosis and prognosis evaluation.

Long non‐coding RNAs (lncRNAs) are a class of non‐coding RNAs with a length of longer than 200 nucleotides.^6^, ^7^ Dysregulation of lncRNAs has been shown to play a critical role in regulating the initiation and progression of BC.^8^, ^9^ For instance, OLBC15 was highly expressed in triple‐negative BC, and OLBC15 promoted the BC tumorigenesis through destabilizing ZNF326.^10^ Exosomes are 30–100 nm nanovesicles containing many molecules, such as lncRNAs, proteins, DNA, RNA, and miRNAs.^11^, ^12^, ^13^ Exosomal lncRNAs are correlated with cancer tumorigenesis and progression and can be used as effective biomarkers in various types of cancer including BC. For example, compared to benign breast disease (BBD) and healthy controls, serum exosomal H19 was significantly increased in BC patients, and high serum exosomal H19 expression was strongly associated with worse clinical variables.^14^ Similarly, patients with BC exhibited higher serum exosomal HOTAIR levels, and overexpression of serum exosomal HOTAIR was correlated with poorer prognosis.^15^

LncRNA differentiation antagonizing non‐protein coding RNA (DANCR or ANCR), located on human chromosome 4, has been previously found to be involved in the initiation and the progression of BC.^16^, ^17^ However, the diagnostic and prognostic value of circulating exosomal DANCR in BC has not yet been explored. Thus, this study aimed to detect the serum exosomal lncRNA DANCR levels in BC patients, BBD patients, and normal controls and analyzed its role as a potential novel biomarker for BC diagnosis and prognosis.

## 2 MATERIALS AND METHODS

### 2.1 Patients and samples

The current study was approved by the Ethics Committee of Taizhou Municipal Hospital. Written informed consent was obtained from all patients and healthy subjects. In this study, blood samples were obtained from 120 BC patients, 70 BBD patients, and 105 healthy volunteers. All recruited BC patients had not received any chemotherapy or radiotherapy prior to the sampling. We also collected the blood samples from all BC cases one month after their surgery. The blood samples were collected in anti‐coagulative tubes and centrifuged at 1600 *g* for 10 min, followed by centrifugation at 16,000 *g* for 10 min at 4°C. The supernatant was stored at −80°C until further use. The clinical characteristics of all 120 patients with BC are summarized in Table 1.

**TABLE 1:** Association of serum exosomal lncRNA DANCR with clinicopathological characteristics

Parameters	Cases	Serum exosomal lncRNA DANCR		*p*
		Low	High	
Age				
<50	49	28	21	0.1936
≥50	71	32	39	
Tumor size (cm)				
<3	66	37	29	0.1421
≥3	54	23	31	
PR status				
Positive	57	33	24	0.0999
Negative	63	27	36	
LN metastasis				
Negative	44	29	15	0.0080
Positive	76	31	45	
ER status				
Positive	47	30	17	0.0150
Negative	73	30	43	
HER2 status				
Positive	39	28	11	0.0009
Negative	81	32	49	
TNM stage				
I/II	77	51	26	<0.0001
III/IV	43	9	34	

### 2.2 Exosome isolation

Total exosomes were isolated from serum with the total exosome isolation reagent (Invitrogen) according to the manufacturer's protocol. The serum was thawed on ice at 25°C. Then, possible residual cell debris were removed by a centrifugation step at 2000 *g* for 30 min. Then, serum supernatant was mixed with the exosome isolation reagent, followed by incubation for at 4°C 30 min and centrifugation at 10,000 *g* for 10 min. The supernatant was discarded, and the exosomal pellet was resuspended in PBS for RNA extraction.

### 2.3 RNA extraction and quantitative real‐time reverse transcription‐polymerase chain reaction (qRT‐PCR)

Total exosomal RNA was extracted from serum samples with an miRNeasy Serum/Plasma Kit (QIAGEN). The RNA was quantified on a NanoDrop ND‐1000 Spectrophotometer (Thermo Scientific) and immediately reverse transcribed into cDNA. Before RNA extraction, 25 fmol/ml of synthesized cel‐miR‐39 (Applied Biosystems) was added as an exogenous spike in control. Then, qRT‐PCR was performed with SYBR PrimeScript miRNA RT‐PCR kit (Takara Biotechnology Co. Ltd) on the 7500 Real‐Time PCR systems (Applied Biosystems). The relative levels of serum exosomal lncRNA DANCR were expressed using the 2^–ΔΔCt^ method.

### 2.4 Measurement of CA153 and CEA in serum

The levels of CA153 and CEA were measured using electrochemiluminescence assays with available commercial kits.

### 2.5 Statistical analysis

The Mann‐Whitney U and Kruskal‐Wallis tests were used to compare the difference regarding the serum exosomal lncRNA DANCR expression between two or more groups. The chi‐square test was performed to evaluate the associations between clinicopathological variables and serum exosomal lncRNA DANCR expression. Receiver operating characteristic (ROC) curves and the area under the curves (AUC) were applied to access diagnostic power of serum exosomal lncRNA DANCR, CA153, and CEA in BC. Kaplan‐Meier analysis plus log‐rank test was used for overall survival (OS) analysis. Multivariate Cox proportional hazard models were used to analyze the independent risk indicators for BC patients. All statistical analyses were performed using GraphPad Prism 6.0 (GraphPad Software Inc.). *p* < 0.05 was considered statistically significant.

## 3 RESULTS

### 3.1 Serum exosomal lncRNA DANCR was dramatically increased in BC patients

**FIGURE 1:**
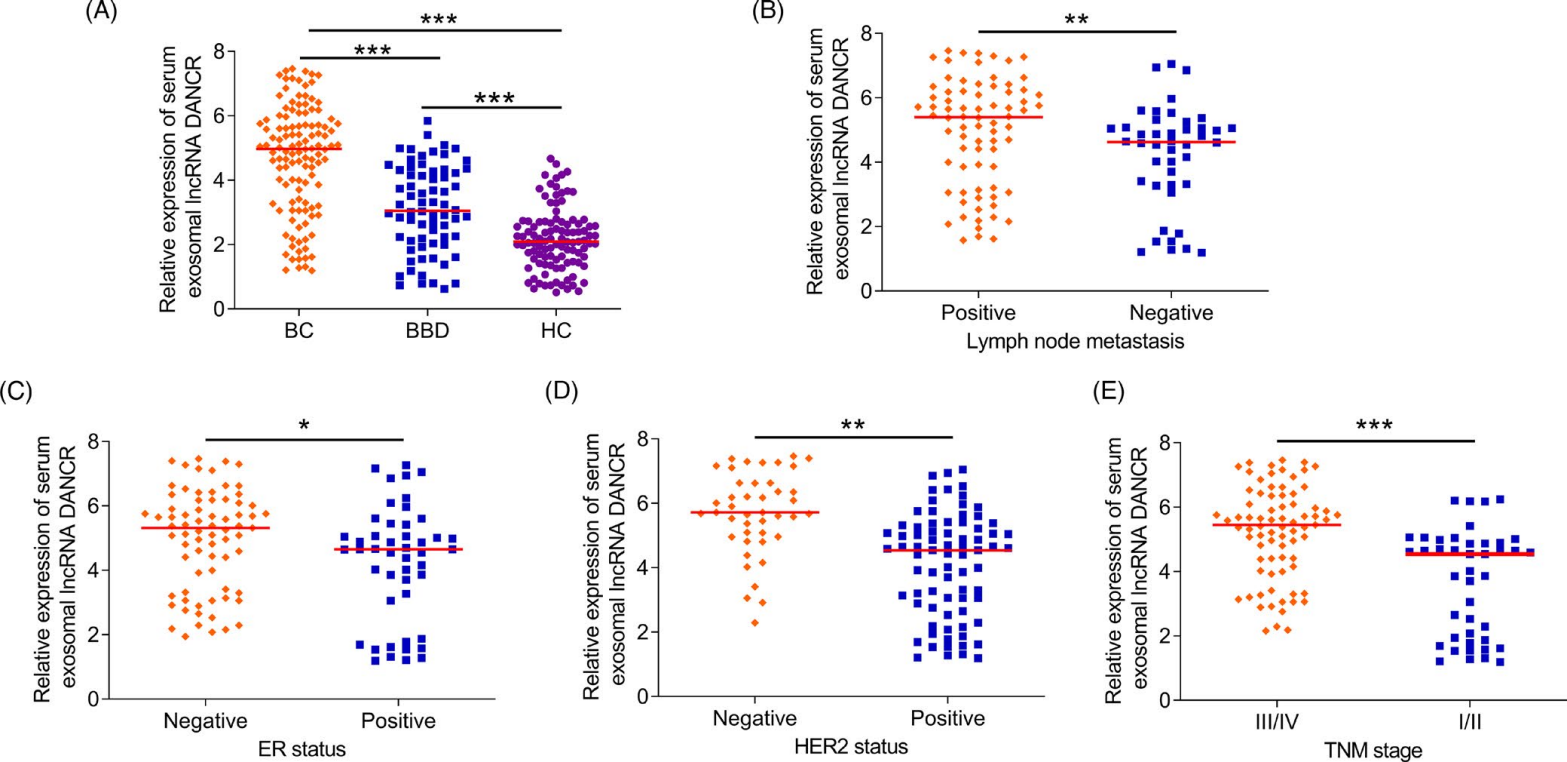
Serum exosomal lncRNA DANCR levels were significantly higher in BC patients (A). Increased serum exosomal lncRNA DANCR expression occurred more frequently in BC patients with positive lymph node metastasis (B), negative ER status (C), negative HER2 status (D), and advanced TNM stage (E)

qRT‐PCR was used to detect the serum exosomal lncRNA DANCR levels in all participants. Serum exosomal lncRNA DANCR levels were significantly higher in BC patients than in BBD subjects and controls (*p* < 0.001, Figure 1A). In addition, BC patients with positive lymph node metastasis (*p* = 0.005, Figure 1B), negative ER status (*p* = 0.012, Figure 1C), negative HER2 status (*p* < 0.001, Figure 1D), and advanced TNM stage (*p* < 0.001, Figure 1E) exhibited higher serum exosomal lncRNA DANCR levels compared with their respective controls.

### 3.2 Diagnostic accuracy of serum exosomal lncRNA DNACR for BC

**FIGURE 2:**
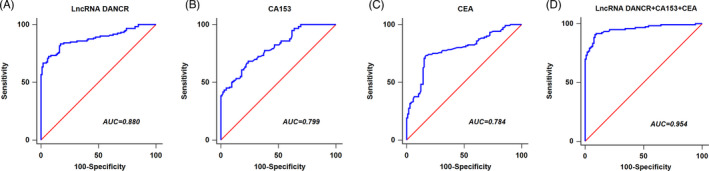
Receiver operating characteristic (ROC) curves of single and combining three markers with the optimum cut‐off values in differentiating breast cancer (BC) from controls

By ROC analysis, Figure 2A demonstrated that the AUC for serum exosomal lncRNA DANCR was 0.880, with a specificity of 82.9% and a sensitivity of 83.3%. CA153 and CEA yielded an AUC of 0.799 (specificity: 76.0%; sensitivity: 68.3%) and 0.784 (specificity: 83.8%, sensitivity: 72.5%) for discriminating BC cases from controls (Figure 2B,C). More importantly, the combination of serum exosomal lncRNA DANCR, CA153, and CEA presented further improvement with an AUC of 0.954 (specificity: 91.4%, sensitivity: 90.8%) (Figure 2D; Table 2).

**TABLE 2:** The diagnostic value of all individual and combined biomarkers for BC

Biomarker	AUC	95% CI	Specificity (%)	Sensitivity (%)
DANCR	0.880	0.800–0.898	82.9	83.3
CA153	0.799	0.740–0.849	76.0	68.3
CEA	0.784	0.725–0.836	83.8	72.5
DANCR + CA153 + CEA	0.954	0.918–0.978	91.4	90.8

Abbreviations: AUC, area under the curves; BC, breast cancer; CA153, Cancer antigen 153; CEA, carcinoembryonic antigen; DANCR, differentiation antagonizing non‐protein coding RNA.

### 3.3 Increased serum exosomal lncRNA DANCR expression was correlated with clinical variables in BC patients

The median serum exosomal lncRNA DANCR expression was used as a cut‐off value, and all 120 BC patients were classified into two groups: high serum exosomal DANCR expression group (*n* = 60) and low serum exosomal DANCR expression group (*n* = 60). As illustrated in Table 1, high serum exosomal lncRNA DANCR expression occurred more frequently in BC cases with positive lymph node metastasis (*p* = 0.0080), negative ER status (*p* = 0.0150), negative HER2 status (*p* = 0.0009), and advanced TNM stage (*p* < 0.0001). However, there was no significant correlation between serum exosomal lncRNA DANCR expression and age, tumor size, and PR status (all *p* > 0.05).

### 3.4 Alteration of serum exosomal lncRNA DANCR levels following treatments

**FIGURE 3:**
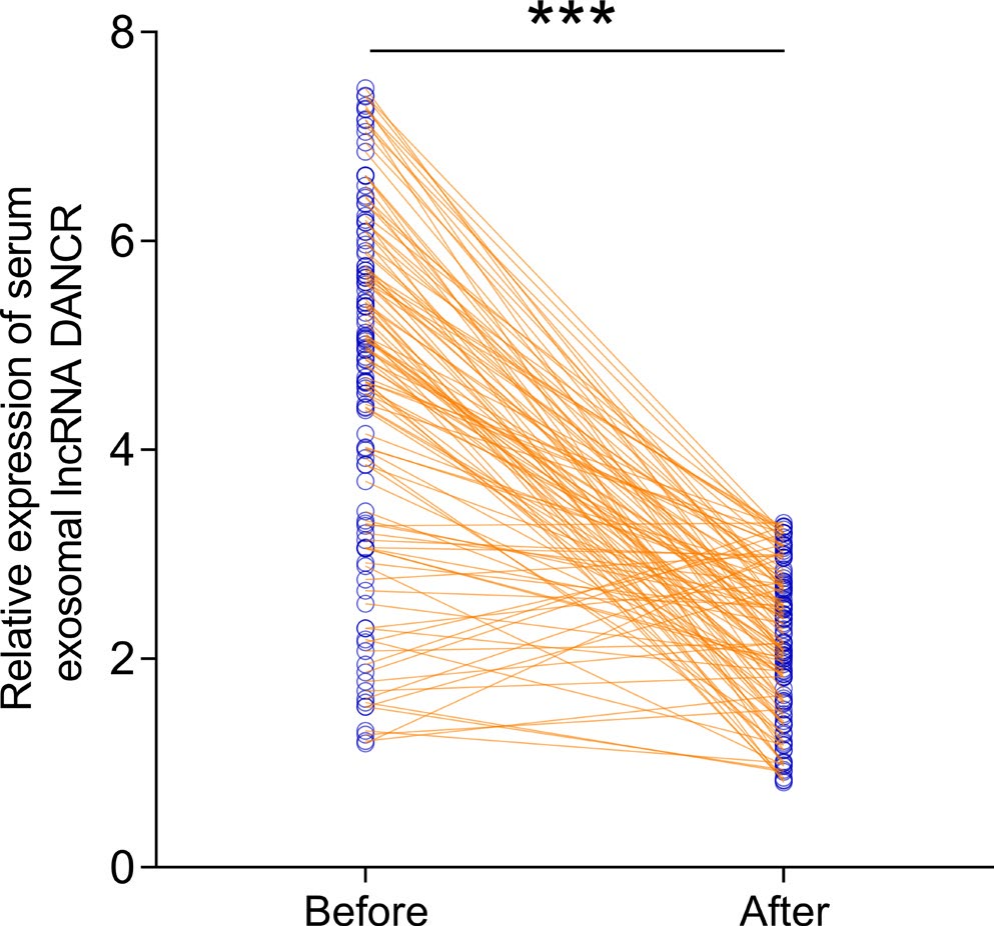
Compared to preoperative blood samples, serum exosomal long non‐coding RNAs (lncRNAs) differentiation antagonizing non‐protein coding RNA (DANCR) levels were markedly downregulated in postoperative samples

One month after surgical resection, paired blood samples were collected from all BC subjects. qRT‐PCR was used to measure the serum exosomal lncRNA DANCR expression level, and we found the serum exosomal lncRNA DANCR levels were markedly reduced following surgery (*p* < 0.001, Figure 3).

### 3.5 Serum exosomal lncRNA DANCR expression was a prognostic biomarker for BC

**FIGURE 4:**
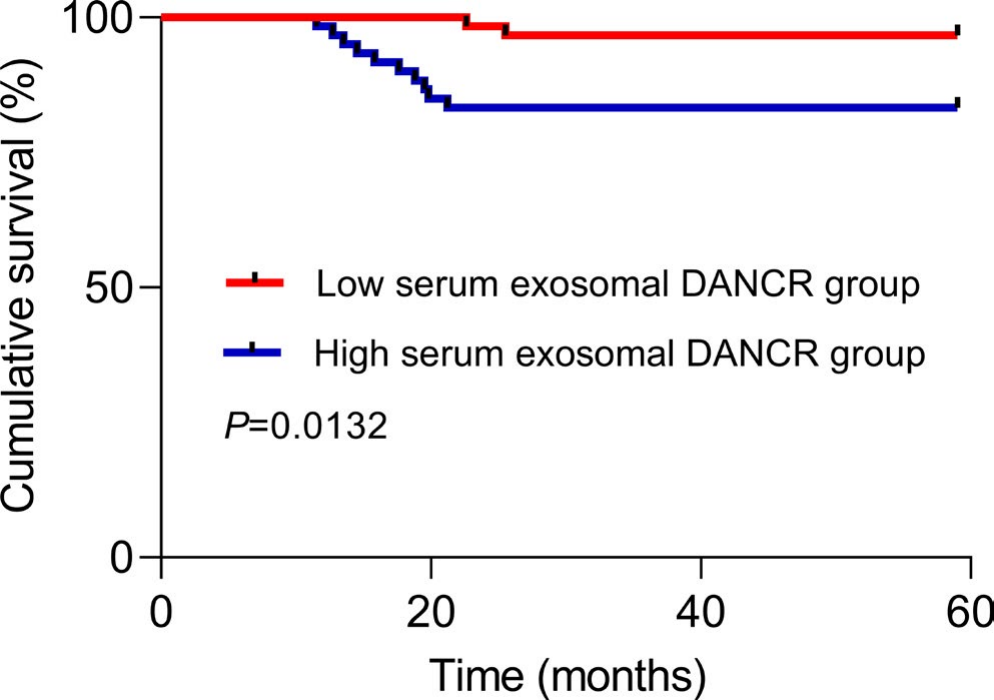
Kaplan‐Meier analysis of overall survival (OS) according to serum exosomal long non‐coding RNAs (lncRNAs) differentiation antagonizing non‐protein coding RNA (DANCR) expression

The Kaplan‐Meier curve for OS regarding serum exosomal lncRNA DANCR expression is presented in Figure 4. Compared to BC patients with low serum exosomal lncRNA DANCR expression, patients with high serum exosomal lncRNA DANCR survived significantly shorter (*p* = 0.0132). Furthermore, multivariate analysis indicated that lymph node metastasis (HR = 3.12, 95% CI = 1.65–5.78, *p* = 0.018), ER status (HR = 2.75, 95% CI = 1.38–5.26, *p* = 0.021), HER2 status (HR = 3.51, 95% CI = 1.92–6.24, *p* = 0.014), TNM stage (HR = 4.75, 95% CI = 2.74–8.32, *p* < 0.001), and serum exosomal lncRNA DANCR expression (HR = 3.86, 95% CI = 2.16–6.63, *p* = 0.009) were significant independent prognostic markers for shorter OS (Table 3).

**TABLE 3:** Multivariate analysis of overall survival in 120 BC patients

Variables	Hazard ratio	95% CI	*P*
Lymph node metastasis	3.12	1.65–5.78	0.018
ER status	2.75	1.38–5.26	0.021
HER2 status	3.51	1.92–6.24	0.014
TNM stage	4.75	2.74–8.32	<0.001
DANCR	3.86	2.16–6.63	0.009

Abbreviations: BC, breast cancer; DANCR, differentiation antagonizing non‐protein coding RNA.

## 4 DISCUSSION

Dysregulation of lncRNAs has been demonstrated to play major roles in breast cancer progression.^18^ This study enrolled a total of 120 BC patients, 70 BBD patients, and 105 healthy women. Compared to BBD patients and controls, a significantly higher serum exosomal lncRNA DANCR level was observed in BC patients. High serum exosomal lncRNA DANCR expression is strongly associated with aggressive clinical variables. In addition, serum exosomal lncRNA DANCR had a relatively high diagnostic performance than CA153 and CEA. The combination of serum exosomal lncRNA DANCR, CA153, and CEA significantly improved the diagnostic accuracy. Moreover, serum exosomal lncRNA DANCR levels were markedly decreased 1 month after surgery. Furthermore, high serum exosomal lncRNA DANCR predicted unfavorable OS in BC patients and was an independent prognostic indicator for BC. The results have demonstrated that serum exosomal lncRNA DANCR is a promising and robust biomarker for BC.

Our findings were in line with several previous studies. Zhang et al. showed that lncRNA DANCR was significantly upregulated both in BC tissues and cell lines. Overexpression of DANCR stimulated epithelial‐mesenchymal transition (EMT), cancer stemness, inflammation, and vice versa. The oncogenic activities of DANCR were reversed by EZH2 inhibition or SOCS3 upregulation.^16^ Similarly, DANCR was significantly higher in BC tissues than in paired normal tissues. DANCR upregulation not only promoted tumorigenicity in xenograft animal but also greatly enhanced cell proliferation, invasion, and migration in vitro through regulating miR‐216a‐5p.^17^

Besides BC, the role of lncRNA DANCR was also reported in different types of cancer. LncRNA DANCR was increased both in tumor tissues and serum samples of gastric cancer (GC) patients, and its upregulation was closely associated with malignant phenotypes. LncRNA DANCR overexpression significantly promoted the carcinogenesis in vitro and in vivo, while inhibition of DANCR showed the opposite effect.^19^ In bladder cancer (BC), lncRNA DANCR expression was dramatically higher in BC tissues than in normal controls. LncRNA DANCR functioned as a miR‐149 sponge to positively correlate with MSI2 expression and markedly stimulated the tumorigenicity. High DANCR expression was strongly associated with aggressive clinical variables patients with BC, such as lymph node metastasis and advanced TNM stage.^20^, ^21^ In addition, lncRNA DANCR was highly expressed in colorectal cancer (CRC) tissues compared to normal tissues, and its overexpression was negatively associated with CRC patient survival. Upregulation of lncRNA DANCR significantly promoted carcinogenesis and metastasis via sponging miR‐577.^22^, ^23^ Moreover, lncRNA DANCR was remarkably increased in ovarian cancer (OC) tissues and cell lines. Overexpression of lncRNA DANCR significantly promoted OC cell proliferation, invasion, and migration through regulating IGF2 or UPF1.^24^, ^25^ However, lncRNA DANCR expression was frequently lower in papillary thyroid cancer (PTC) tissues than that in adjacent normal tissues, and low lncRNA DANCR expression was closely associated with worse clinical parameters of PTC patients, suggesting that lncRNA DANCR played a tumor suppressive role in PTC.^26^ The contradictory functions of lncRNA DANCR may be explained by its ambiguous role in regulation of the complex networks of oncogenes and tumor suppressors resulting in a cancer type‐dependent outcome.

## 5 CONCLUSIONS

Taken together, this study demonstrated that serum exosomal lncRNA DANCR expression was markedly elevated in patients with BC. Upregulation of serum exosomal lncRNA DANCR was closely associated with worse clinical parameters and shorter survival. Thus, serum exosomal lncRNA DANCR serves as a promising prognostic marker for BC. Meanwhile, as a result of small sample research, the analysis of larger cohorts is required to confirm the clinical role of serum exosomal lncRNA DANCR in BC.

## CONFLICT OF INTERESTS

None.

## Data Availability

The datasets used and/or analyzed during the current study are available from the corresponding author on reasonable request.
